# Effects of expectation on face perception and its association with expertise

**DOI:** 10.1038/s41598-024-59284-0

**Published:** 2024-04-24

**Authors:** Inês Mares, Fraser W. Smith, E. J. Goddard, Lianne Keighery, Michael Pappasava, Louise Ewing, Marie L. Smith

**Affiliations:** 1 School of Psychological Sciences, Birkbeck College, University of London, London, UK; 2 William James Center for Research, Ispa – Instituto Universitário, Lisbon, Portugal; 3 School of Psychology, University of East Anglia, Norwich, UK; 4 Centre for Genomics and Child Health, Blizard Institute, Queen Mary University of London, London, UK; 5 Centre for Brain and Cognitive Development, Birkbeck College, University of London, London, UK; 6 Department of Clinical and Movement Neurosciences, Queen Square Institute of Neurology, University College London, London, UK

**Keywords:** Expectation, Prediction, Expertise, Face processing, EEG, MVPA, Perception, Visual system

## Abstract

Perceptual decisions are derived from the combination of priors and sensorial input. While priors are broadly understood to reflect experience/expertise developed over one’s lifetime, the role of perceptual expertise at the individual level has seldom been directly explored. Here, we manipulate probabilistic information associated with a high and low expertise category (faces and cars respectively), while assessing individual level of expertise with each category. 67 participants learned the probabilistic association between a color cue and each target category (face/car) in a behavioural categorization task. Neural activity (EEG) was then recorded in a similar paradigm in the same participants featuring the previously learned contingencies without the explicit task. Behaviourally, perception of the higher expertise category (faces) was modulated by expectation. Specifically, we observed facilitatory and interference effects when targets were correctly or incorrectly expected, which were also associated with independently measured individual levels of face expertise. Multivariate pattern analysis of the EEG signal revealed clear effects of expectation from 100 ms post stimulus, with significant decoding of the neural response to expected vs. not stimuli, when viewing identical images. Latency of peak decoding when participants saw faces was directly associated with individual level facilitation effects in the behavioural task. The current results not only provide time sensitive evidence of expectation effects on early perception but highlight the role of higher-level expertise on forming priors.

## Introduction

Expectations about the world around us are well known to influence sensory processing in both early and higher-level brain areas and may critically support our ability to address the computational demands posed by a dynamic, ever-changing world^1–3^. By extracting statistical regularities from past and present sensorial input, we can build internal models to generate predictions regarding the nature of incoming sensorial information which guide subsequent perception^4,5^. Indeed, there is an increasing body of work that demonstrates the role of probabilistic priors in shaping perception^6–8^. Predictive coding is a prominent computational theory that provides a framework to interpret such expectancy effects. Within this framework, perception is proposed to be dynamically generated through a process of iterative comparison between sensory inputs and prior predictions^1,3^. Each hierarchical processing stage is suggested to contain both representational units that encode the probability of a stimulus occurring (the expectation) and those that encode any mismatch between the prediction and the bottom-up sensory inputs (prediction errors/surprise^3^). The latter prediction errors are then thought to be propagated via forward connections to higher-level areas allowing representations, i.e. expectations, to be hierarchically updated with this new information^3^.

Studies probing the effect of expectation on the neural and behavioural markers of perception often manipulate expectations in an artificial manner, an approach that cannot capture the impact of real-life experience on forming probabilistic priors. Exceptions to this have either (1) explored violations of naturally occurring life learned expectations (e.g. illumination of the world from above, or canonical object orientation^9–11^), or (2) explored the role of experience at the categorical level by contrasting responses associated with a category of particular expertise with a non-expert category (e.g. the roman alphabet, familiar visual-orthographic characters or faces^12^ vs false fonts^13^; non familiar characters^14^; other objects^15^; other race faces^16^). These effects of expectation are sometimes considered in the context of fMRI repetition suppression, a phenomenon whereby repeated stimuli elicit decreased neural activation. Effects of expectation (i.e., effects of the probability of repetition on RS) have been selectively observed for participants’ category of expertise and not for non-expert conditions^12,15^. Such findings suggest that increased experience and expertise with a particular stimulus category may allow participants to generate more robust predictions that selectively modulate activity in visual areas. This line of research relies on contrasting responses to different visual stimuli. Yet this approach precludes firm conclusions about effects of expertise which can be driven by differences inherent to the stimuli category such as differential attention capture. An alternative would be to consider how an individual’s pre-existing expertise in a given category domain can have an effect on their ability to form expectations. This approach has rarely been the focus of targeted research.

In the current study we investigate expertise effects naturalistically, by harnessing the inherent and well described variability in face processing expertise across the population^17^. While face perception is ubiquitous, the underlying processing skills are known to vary greatly in the general population. There is a well-defined normal distribution of face identification ability ranging from individuals considered to be super-recognizers^17^, to individuals with developmental prosopagnosia that struggle to recognize even close friends and relatives^18^. Considerable research interest has been given to the mechanisms underlying these individual differences^18^, with the effect of lifelong experience being known to influence the development of face abilities. This is evidenced by other-race effects^19–21^, and hometown size effects^22–24^ (see^18^ for a review of other potential contributions). Importantly, face processing ability is supported by a specialized extended network (e.g. Fusiform face area, FFA, Superior Temporal Sulcus, STS, anterior temporal face patch, among others) shown to be sensitive to face expectation across multiple studies^25–28^ (see^29^ for a review). Yet, evidence that participants’ level of face experience/expertise modulates such expectation-driven perception is scarce. One exception is an fMRI study investigating the effects of autistic traits upon neural responses to face stimuli. This study reported a correlation between repetition suppression (a marker of prediction error) and a behavioural measure of face memory in regions of the left parietal and prefrontal cortex across all participants^30^.

Here we seek to further explore the idea that face processing expertise at the individual level will be associated with the ability to generate more robust predictions when a face is expected. This would result in increased neural activation and concomitant behavioural interference when faces are highly expected but not observed (i.e., a different visual object is presented instead). Such an outcome would be in line with studies showing that stimulus expectation is sufficient to elicit activation of the corresponding template in early sensorial areas (V1) even in the absence of the stimulus itself^31^. Thus, in the current study, we systematically manipulate expectations about stimuli from a category of expertise (faces), and a well matched less-expert control object category (cars), on a trial-by-trial basis. An independent task, the Cambridge Face Memory Test—long version (CFMT+^17^) will provide an independent assessment of face expertise at the individual level.

Predictive coding accounts of face perception have previously been probed by measuring neural responses (fMRI activation) after manipulating stimulus expectation with a preceding colour cue^26^. Similar levels of activity in the FFA were observed for face and object (house) stimuli when there was high face expectation, but maximal activity differentiation was found when face expectation was low (high face surprise for face stimuli, but not for houses). This stands in contrast to traditional perceptual accounts that would predict consistently stronger responses to faces than another object category in this face selective neural region. These findings suggest that neural responses are driven both by expectation and surprise associated with the expert category (faces). Here we will employ a similar paradigm, where participants learn to associate a particular colour cue with an object category (namely cars), such that the cue is predictive 75% of the time for faces or cars. We anticipate that responses associated with these complementary components of predictive coding—expectation and surprise– will be selectively enhanced for the category of expertise (faces) compared to our control category (cars).

First, in a behavioral task participants will be trained to learn the probabilistic contingency between a colour cue and target stimulus category leading to trials with high and low probabilistic face expectation (for clarity, this corresponds to low and high car expectation respectively). High face expectation is predicted to facilitate categorization of faces and interfere with categorization of cars, both associated with participants’ individual level of face expertise. Similarly, in a subsequent EEG task using the same learned associations, increased neural responses are predicted for high vs low face expectation when a car is observed (driven by activity of representational neural units predicting a face). In low face expectation trials with a face presented, we predict that decreased activity of face selective representational units will be balanced at a populational level by an increased activity in neural units coding prediction error (i.e. face surprise). Of note, previous research has proposed that face sensitive areas do not code for object surprise, i.e. seeing an unexpected car^26^. The N170 component will be analysed given its well-established face sensitivity^32,33^, and association with prediction errors^9,34–37^. Importantly, in this design, and in these comparisons we will directly analyse the behavioural and neural effects of expectation by contrasting responses to exactly the same visual input (face or car stimulus).

Finally, we will go beyond the standard ERP analysis of single established face related components by decoding the neural response to contingencies of interest. While analysis of specific ERP components can be theoretically informative, it is necessarily limited to a discrete, often short timeframe and a localized region of interest: a few select electrodes. Multivariate pattern analysis (MVPA) permits more comprehensive and thorough exploration of the neural underpinnings of a given phenomenon^38,39^. We predict that MVPA will highlight significant differential decoding of the neural response, over visual posterior areas, to exactly the same visual input when there is high vs low face expectation. We predict that this effect will be particularly present for observed cars, where there is not an effect of face surprise. Furthermore, we predict that the extent of this decoding will be associated with participants’ individual level face expertise.

## Methods

We report how we determined our sample size, all data exclusions (if any), all manipulations, and all measures in the study. Sample size, hypothesis and data analysis were registered beforehand (https://aspredicted.org/; for behavioural task 91635, for EEG task 91638).

### Participants

Sample size was calculated for correlations between 0.3 and 0.5, based on the range of effect sizes observed in a previous study from our group that investigated the neural links (i.e. using ERP and decoding measures) of face expertise^38^. For these effect sizes and a power of 80% (considering *p* < 0.05), between 29 and 84 participants were required^40^. As per our pre-registration, we set a minimum testing goal of 60 participants (and a maximum of 84).

A total of 67 participants took part in the experiment. To avoid known other race biases^41^, only White participants were included (4 who did not meet this criteria were tested but not included). One participant was further excluded due to more than 30% of trials with a reaction time above 1 s (as per the pre-registration). Our final sample of participants included 62 volunteers (age: 27.52 ± 6.24 years; 44 female, 16 male, 2 non-binary; 57 right-handed, 4 left-handed, 1 ambidextrous). All participants reported normal or corrected to normal vision. Participants received a small monetary reimbursement (£8.5 per hour of experiment) or undergraduate course credits, as compensation for their time. Written informed consent was obtained prior to participation. This study obtained ethical approval from the Ethical Committee of the Department of Psychological Sciences, Birkbeck College, University of London (Reference 2223011). All methods were performed in accordance with the Declaration of Helsinki.

### Stimuli

To minimize participant habituation to stimuli, different stimulus images sets were used in the behavioural and EEG tasks. The behavioural task set comprised five female and five male identities with neutral facial expressions^42^ and ten car models. Images of cars were extracted from websites of different brands (only one car model was selected per brand). To increase difficulty and avoid participants automatically categorizing stimuli as faces or cars based on a single difference of outline, different viewpoints were selected. Each identity (face or car) was always presented in the same viewpoint (for each category, 4 identities were shown in a frontal view, 3 turned to the right side and 3 to the left side). All images were converted to greyscale (size: 416 × 496 px). Face stimuli were digitally manipulated to remove any external features such as hairstyle or background and car stimuli similarly had all logotypes and external features such as backgrounds digitally removed. For the EEG task six different identities (3 female, 3 male) from the Chicago face database^43^ were selected. All images were of neutral expression faces, facing forward. A mask provided the same general outline for all face images. Images of six different models of cars, facing forward, were collected and processed in the same manner for use as in the behavioural task. Luminance and contrast were controlled across stimuli for each task using the Shine toolbox^44^.

### Procedure

**Figure 1:**
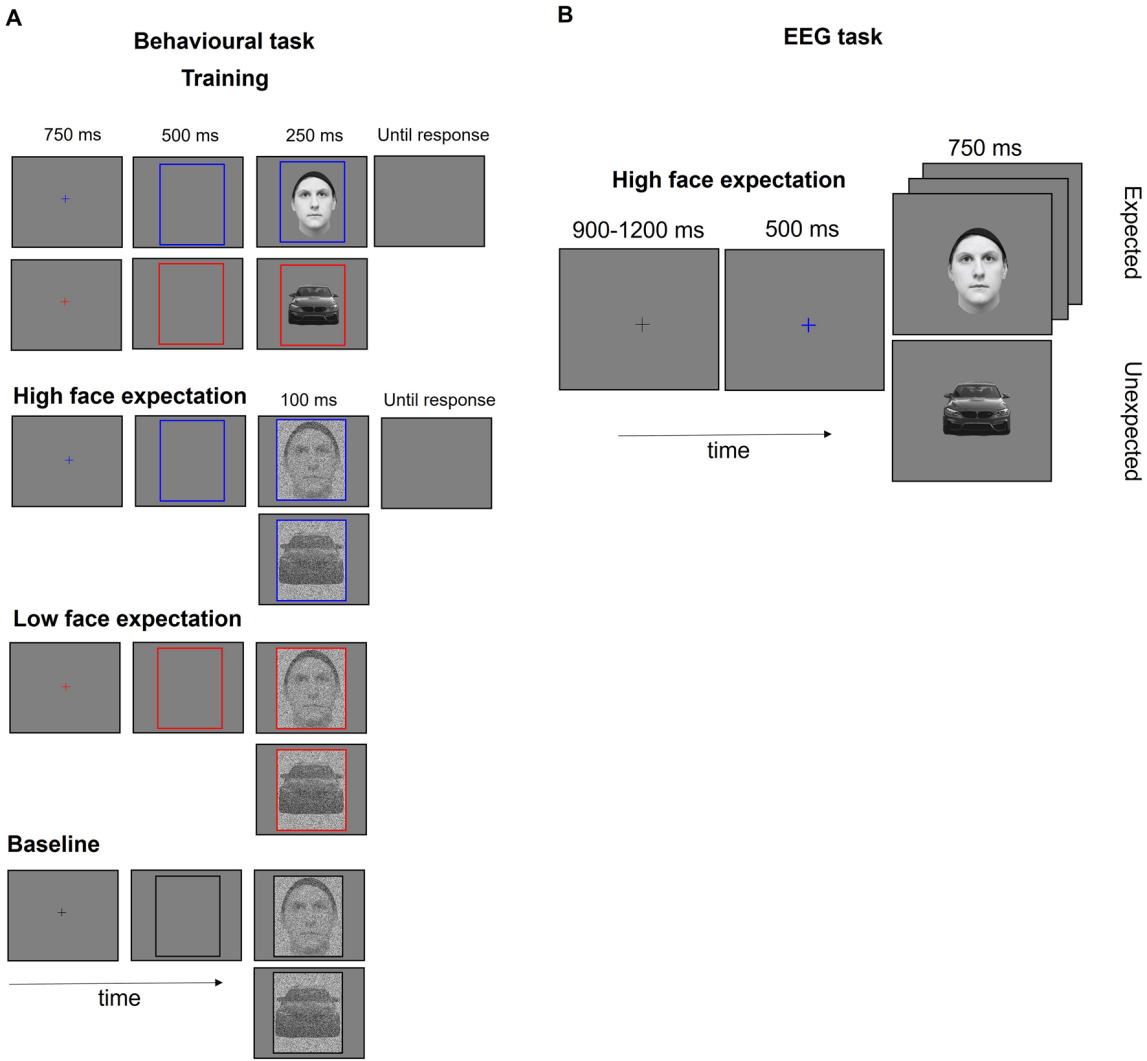
Overall schematics for the task procedure of the (**A**) Behavioral and (**B**) EEG task. Stimuli presented for illustration purposes only. Informed consent was obtained from all subjects and/or their legal guardian(s) for publication of identifying information/images in an online open-access publication.

Participants completed a battery of tasks during an approximately 180 min session including (1) a behavioural task, where participants learnt the association between a colour and an object category (faces or cars), followed by (2) an EEG task where participants observed stimuli of these two categories preceded by a fixation cross matching the learned association or not (Fig. 1 for procedure of the behavioural and EEG tasks). Participants also completed an additional EEG experiment as part of a larger project which will not be reported here. Finally, participants completed (3) a measure of face expertise, the Cambridge Face Memory Test—long form (CFMT+^17^), and (4) a broadly matched measure of object expertise, the Cambridge Car Memory Test (CCMT^45^).

The behavioral association task was presented using the Gorilla.sc platform^46^, with participants sitting at a comfortable distance from a laptop screen. The task had two stages, a training stage and a testing stage. In the training stage participants learned to consistently associate a colour cue (red or blue, counterbalanced between participants) with one of two categories (i.e. faces or cars, 30 trials each, total of 60). Trials started with a predictive cue, a fixation cross with a colour matching the trial category (750 ms), followed by an empty frame of the same colour (500 ms). The target image was then briefly displayed inside the frame for 250 ms. Participants were asked to categorise the image as a face or a car, with a button press. A blank screen remained until participants responded. The training phase ended for all participants upon completion of the 60 trials. In the testing stage, participants completed a similar categorization task. To avoid automatic responses and increase task difficulty, the target image appeared for a shorter period (only 100 ms), and visual noise was added to the images. This was done using the MATLAB function ‘imnoise’, with a density of 0.75 and ‘salt & pepper’ type of noise (on and off pixels). To maintain training effects, the target was consistent with the predictive colour cues in 75% of trials (i.e. 120 trials in each category, 90 trials with the expected image, 30 trials with an unexpected image, making a total of 240 trials). To provide an estimate of category specific baseline performance, a further 60 trials (30 cars and 30 faces), were presented without any learned association. In these trials a black fixation cross and frame, not previously associated with any condition, preceded the target image. Participants completed a total of 300 trials of the test phase over 4 blocks.

For the main EEG task participants sat in an electrically shielded and sound-proof room at a distance of approximately 70 cm from the screen (stimuli subtended approximately 5.3 width by 6.1 height of visual angle; 250 × 295 pixels). This task was presented using the E-prime software (Version 2.0). Participants were asked to carefully attend to the target stimuli (faces and cars) while searching for a letter (an ‘A’ or ‘K’), presented centrally in catch trials (40 trials, around 7.7% of total trials). Trials started with a black fixation cross (duration 900–1200 ms, pre-allocated randomly to the trial list in steps of 25 ms). This was followed by a predictive cue, a fixation cross of a colour associated to one of the stimuli categories (750 ms), and then by the target stimuli (500 ms). As before, to maintain the predictive contingency, the target was consistent with the predictive colour cues in 75% of trials (i.e. 240 trials were presented for each category of which 180 trials displayed an image of the predicted category while 60 trials presented an image of the unexpected category). A total of 520 trials were randomly presented split in 4 blocks to allow for breaks.

To assess participants selective expertise with faces, at the end of the session participants completed the CFMT+^17^ and the CCMT^45^. These recognition memory tasks share a general procedure, whereby participants are introduced to six novel target identities (faces or cars) presented in three different viewpoints. Participants are then presented with a line-up of three similar looking identities (2 foils) and asked to identify the learned identities. The test trials progressively increase in difficulty, adding novel viewpoints and visual noise to the test stimuli, further methodological details are available in the original works^17,45^. Car expertise was measured to allow for tests regarding whether any observed effects of expectation on the behavioural and EEG tasks were directly associated with face-selective recognition ability. To this end we planned to use a more selective measure of face expertise by calculating the residuals from a regression that predicted CFMT+ scores from CCMT scores^47^. Results from the raw scores of the CFMT+ can be seen in supplementary material.

### EEG recording and analysis

EEG signal was recorded using a 64 Ag–AgCl electrode EASYCAP (with electrode placement according to the international 10/10 system). Horizontal eye movements were monitored using two electrodes fitted into the cap (FT9/FT10). Vertical eye movements and blinks were measured using a third electrode placed below the right eye.

Electrode impedance was lowered below 10 μV, and online referenced to FCz (AFz was used as ground). EEG was acquired at a sampling rate of 1000 Hz. Data was analysed using the Matlab (R2020b) toolbox EEGLAB (Version 2022.1^48^). Continuous data was high pass filtered above 0.1 Hz and low pass filtered below 40 Hz (using EEGlab firfilt v2.6 function). Channels were signalled for interpolation using the clean rawdata plugin (v2.7^49^), with criteria for channels to be kept of (1) a maximum length of a flatline of 5 s, (2) a minimum correlation of 0.8 to its calculated reconstruction based on other channels, and (3) smaller line noise relative to its signal than 4 standard deviations calculated based on the total channel population (M = 4.58 channels). Data was then re-referenced to the average reference, and epochs were created around stimulus onset (− 200 to 500 ms, baseline corrected to the 200 ms before stimulus onset). All catch trials were removed from the analysis. Trials with noise were automatically rejected using an amplitude threshold criteria of above 70 or below − 70 μV^50^. The P100 and the N170 components were analysed, as both have shown sensitivity to face stimuli. the P100 component was analysed on channels O1/2 and the N170 on channels P7/8 and PO7/8, averaged in a 40 ms window around each peak (97 and 159 ms respectively).

In addition to standard ERP analysis we further used MVPA to determine if high compared to low face expectation could be distinguished based on brain activity and if this difference was correlated with face expertise. Data was downsampled to 250 Hz, and linear support vector machine (SVM) classifiers were trained for each time sample, based on single trials, using all posterior electrodes (O1/2, P7/8, P3/4, Pz, TP7/8, PO7/8, POz, Oz, PO3/4, P5/6, P1/2, TP9/10). These sites were chosen because previous work has shown that these electrodes contain the most informative signal for visual tasks (see^38,51^). We focused on two binary comparisons where participants saw stimuli from the same visual category (i.e., faces or cars) but face expectation was manipulated (i.e. high vs low).

The classifiers were trained and tested on independent sets of data, with a randomly equalized number of trials per condition. The performance of the classifier was assessed, with a 70% train to 30% test random split of the data, repeated 20 times to form 20 cross-validation iterations see^51^, repeated 100 times for robustness^52^. Accuracy was calculated by testing the trained classifier against the averaged EEG trials of the test set per condition, in order to increase signal to noise ratio^51,53,54^. The same procedure was done on permuted labels to extract a measure of chance level. Averages from the 100 iterations of classifiers and chance level classifiers were calculated. Significant decoding was analysed at the group level via a paired samples t-test, comparing decoding of classifiers and chance level classifiers across all participants (one-tailed) for each time point (False Discovery Rate, FDR, corrected). To limit the number of multiple comparisons, this analysis was only conducted for time samples between 60 and 500 ms (111 comparisons).

To extend this analysis to the individual participant level we sought to establish individual significant decoding. Individual chance level classifiers were extended to include a total of 1000 permutations, creating a null distribution per participant. A classifier using the true labelling was also included in the distribution of results as one of the possible outcomes. Significance at the individual level was calculated as the proportion of the null distribution that was greater than or equal to the accuracy obtained with correct labels, with significance considered when actual decoding is greater than or equal to 95% of the null distribution (FDR corrected, see^38,54,55^). In line with previous research studies applying these methods^38,39,56^ we extracted three metrics: sustainability of decoding—defined as the percentage of significant decoding in a given time-window, peak decoding—defined as the maximal positive peak in a given time window and peak decoding latency—defined as the time-point of the maximal positive peak decoding in a given time-window. These metrics aim to evaluate at the individual level the neural differentiation between the key contrasts as measured through decoding accuracy, both in terms of extent of decoding across the selected time window (60–500 ms), and maximal decoding. One additional metric (decoding onset) was not included here because our expectation-related contrasts are more subtle than those investigated in previous studies (i.e. related to different visual categories). Unsurprisingly this resulted in the absence of significant decoding above values seen at the baseline, in some participants, making it impossible to establish a timepoint for significant decoding onset at the individual level.

## Results

### Behavioural association task

**Figure 2:**
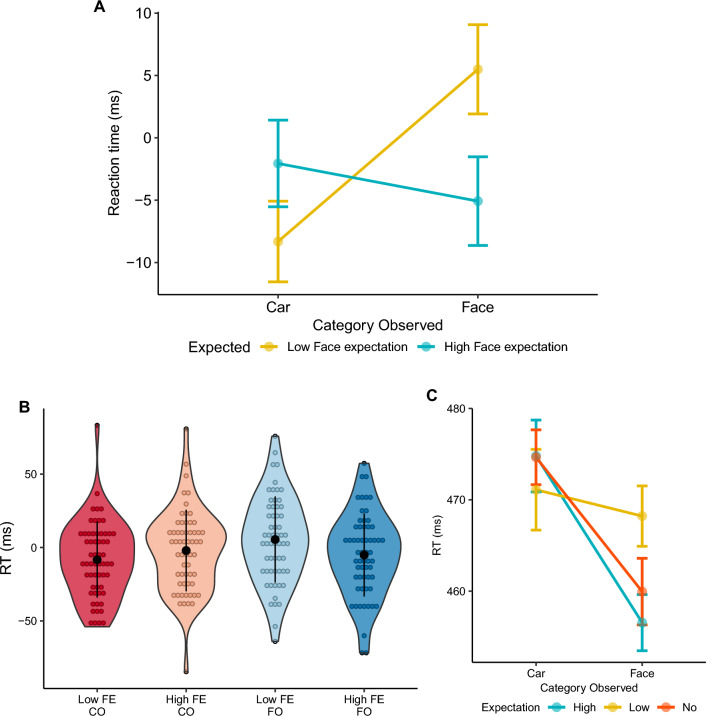
Reaction times shown are corrected by subtracting the mean reaction time of the corresponding baseline condition; (**A**) Behavioral response in face/car stimulus categorization task as a function of level of face expectation. (**B**) Violin plots showing the full distribution of baseline corrected reaction times. FE—Face Expectation; CO—Car Observed; FO—Face observed; (**C**) Non-baseline corrected reaction times for face and car categorization as a function of face expectation for visualization purposes.

Trials with reaction times below 100 ms or above 2 SD relative to individual participants’ mean were removed. Trial numbers were then randomly equalised between conditions to match the condition with the minimum number of trials per participant. Accuracy levels on this task, as originally predicted, were at ceiling 95.52 ± 5.95% precluding us from further analysing this variable. Reaction time was analysed using only correct trials. High and low face expectation trials for observed face and car conditions, were baseline corrected to remove baseline categorical differences in reaction time (${M}_{D}=14.71$, 95% CI $\left[4.59,24.83\right]$, $t\left(61\right)=2.91$, $p=.005$). This was done by subtracting the mean reaction time of the corresponding baseline condition. Note that negative values suggest a facilitation compared to baseline driven by expectation or surprise, while positive values suggest interference. We hypothesized that the incorrect expectation of a category of high expertise (faces), would interfere with categorizing the subsequent unexpected target (car). Given the proposed role of expertise we further hypothesized that the incorrect expectation of a category of low expertise (cars), would not lead to a similar effect on unexpected faces. A two-way repeated measures ANOVA was conducted, with factors of face expectation (2 levels, high vs low; where low face expectation corresponds to car expectation) and observed category (2 levels, face vs car) as within subject variables (Fig. 2). Three participants were removed from this analysis due to unexpected and substantial outlier scores (unexpected at time of pre-registration) having a baseline corrected reaction time more or less than two times the interquartile range. No main effects were observed (Expectation, $F\left(1,58\right)=0.91$, $MSE=302.02$, $p=.345$, ${\widehat{\eta }}_{p}^{2}=.015$; Observed Category $F\left(1,58\right)=1.28$, $MSE=1,342.62$, $p=.263$, ${\widehat{\eta }}_{p}^{2}=.022$) but instead a significant interaction between Expectation and Observed category was identified $F\left(1,58\right)=8.75$, $MSE=477.47$, $p=.004$, ${\widehat{\eta }}_{p}^{2}=.131$. Support for our initial hypothesis that incorrectly expecting a stimulus from a high expertise category would have a particularly detrimental effect on categorization speed was equivocal. This was analysed with two critical contrasts, a) high face expectation trials with a car observed vs. low face expectation trials with face observed, ${M}_{D}=-7.55$, 95% CI $\left[-18.37,3.28\right]$, $t\left(58\right)=-1.40$, $p=.168$; and b) car observed trials, when participants had a high vs low expectation of seeing a face; ${M}_{D}=6.26$, 95% CI $\left[-0.39,12.90\right]$, $t\left(58\right)=1.88$, $p=.064$.

A more robust effect of face expectation (high vs low) was found on trials where faces were observed ${M}_{D}=10.57$, 95% CI $\left[2.71,18.42\right]$, $t\left(58\right)=2.69$, $p=.009$. See Fig. 2.

Exploratorily and to clarify if this effect was due to a facilitation of high face expectation or to an interference of low face expectation, we directly contrasted the effect of each of these conditions with the corresponding baseline trials (no expectation). Expectation-related differences on face observed trials were not solely driven by facilitation or interference of expectation, but by both factors combined (facilitation, ${M}_{D}=-5.08$, 95% CI $\left[-12.45,2.30\right]$, $t\left(58\right)=-1.38$, $p=.174$; interference, ${M}_{D}=5.49$, 95% CI $\left[-2.15,13.13\right]$, $t\left(58\right)=1.44$, $p=.155$).

There was no overall advantage of correct expectation of faces when compared to trials correctly associated with car expectation (i.e., low face expectation; ${M}_{D}=3.24$, 95% CI $\left[-7.07,13.54\right]$, $t\left(58\right)=0.63$, $p=.532$).

**Figure 3:**
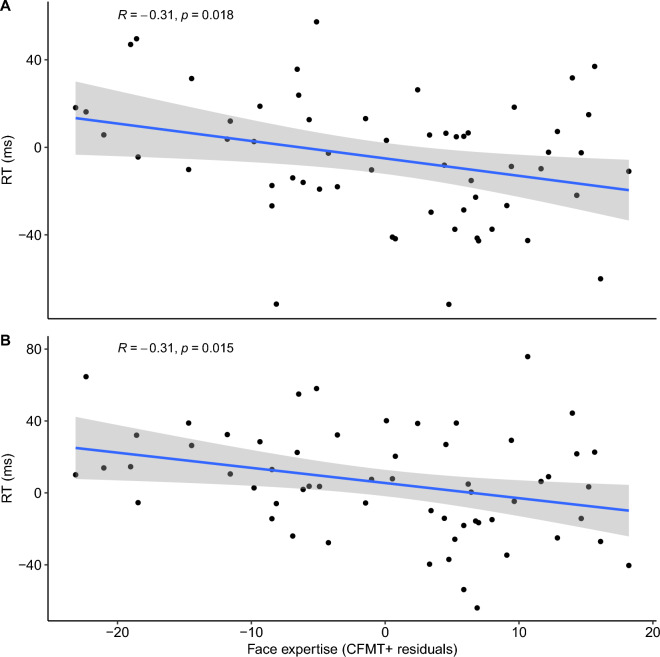
Correlation scatterplots showing the significant association between face expectation effects when participants saw the higher expertise category (faces) and a face-selective measure of expertise. Facilitation effects, (i.e. facilitation of the correct expectation) are shown in panel **A** and interference effects (i.e. interference of the incorrect expectation) on panel **B**.

At the individual level, face expertise (residual CFMT+ scores considering CCMT performance; Supplementary Table 1, for correlation with raw CFMT+) was associated with increased behavioural facilitation when face expectation was high ($r=-.31$, 95% CI $\left[-.52,-.06\right]$, $t\left(57\right)=-2.44$, $p=.018$), and decreased interference when face expectation was low ($r=-.31$, 95% CI $\left[-.53,-.06\right]$, $t\left(57\right)=-2.50$, $p=.015$). See Fig. 3 for scatter plot visualizing this relationship for facilitation (top) and interference (bottom). Importantly, the calculation of these expertise measures accounts for baseline differences in face detection, making these results directly tied to participants’ face expectations. Critically, when this baseline condition is examined independently, there is no correlation between face expertise and RTs $r=.03$, 95% CI $\left[-.23,.28\right]$, $t\left(57\right)=0.21$, $p=.835$. Furthermore there is no correlation between car expertise and any effects of expectation, even for car observed trials (*r* > − 0.13; *p* < 0.325).

### ERP results

Participants with less than 20 trials per condition were removed from the ERP analysis (8 participants were excluded) as per the pre-registration. After any trial-based exclusions due to measurement artifacts, we ensured trial numbers were balanced between face expectation conditions for each participant by removing trials (random selection) to match the condition with fewer trials. For each component, a 2 × 2 × 2 within subject ANOVA was conducted with face expectation (high vs low), category observed (face vs car), and hemisphere (left vs right) as factors.

For the P100, there was no main effect of expectation $F\left(1,53\right)=0.32$, $MSE=2.89$, $p=.572$, ${\widehat{\eta }}_{p}^{2}=.006$, category $F\left(1,53\right)=0.53$, $MSE=4.41$, $p=.471$, ${\widehat{\eta }}_{p}^{2}=.010$, hemisphere $F\left(1,53\right)=0.16$, $MSE=9.49$, $p=.688$, ${\widehat{\eta }}_{p}^{2}=.003$, nor any interactions with expectation effects (*p* < 0.118).

Similarly, for the N170, there was no main effect of expectation $F\left(1,53\right)=0.09$, $MSE=2.20$, $p=.768$, ${\widehat{\eta }}_{p}^{2}=.002$ nor any interactions with expectation (*p* < 0.416). For completeness, in line with previous literature, a main effect of hemisphere $F\left(1,53\right)=9.72$, $MSE=25.35$, $p=.003$, ${\widehat{\eta }}_{p}^{2}=.155$, and category were found $F\left(1,53\right)=61.18$, $MSE=6.80$, $p<.001$, ${\widehat{\eta }}_{p}^{2}=.536$. Hemisphere effects were characterized by an increased N170 for the right hemisphere (M = − 5.34 ± 5.64) compared to left (M = − 3.83 ± 4.56), and category effects category effects by an increased N170 for faces (M = − 5.57 ± 5.12) compared to cars (M = − 3.61 ± 5.07). For the ERP waveforms please see Fig. 1 in supplementary material.

### MVPA decoding results

**Figure 4:**
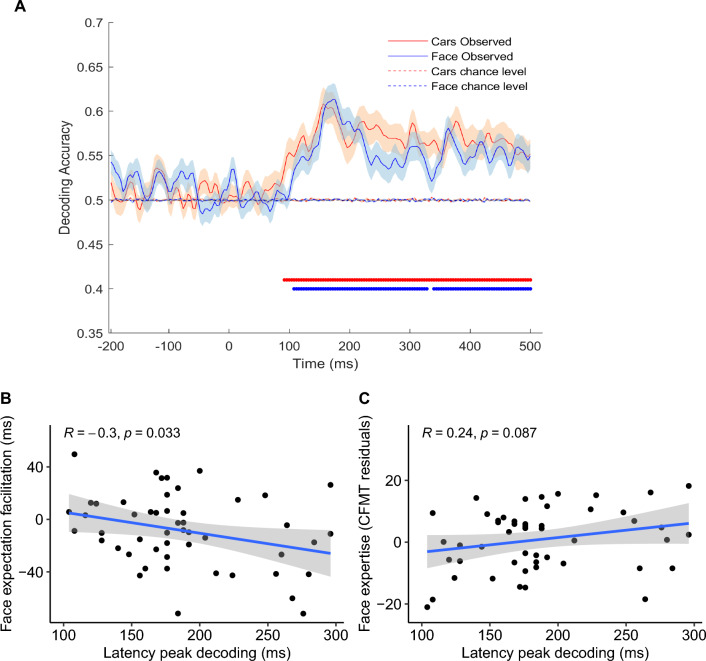
(**A**) Accuracy of MVPA decoding of face expectation (high vs low) when participants observe a category of high expertise (faces, blue line), or low expertise (cars, red line) compared to chance level decoding using random labels. Significant time points are indicated by colour coded dots at the base of the curves. (**B**) Correlation of latency of expectation peak decoding to observed faces and facilitation effects of face categorization in the behavioural. (**C**) Marginal correlation of latency of peak decoding and independent estimation of face expertise.

In line with the pre-registration, participants were only included in each of the two key MVPA contrasts (high vs low face expectation when seeing a car, and high vs low face expectation when seeing a face), when they had more than 30 trials per condition (face observed N = 52; car observed N = 46). Expectation effects were observed from the neural responses when decoding high vs low face expectation for observed car stimuli. Significant decoding started from 92 ms onward until the end of the epoch (500 ms), highlighting differential brain activation associated with face expectation, that critically cannot be explained by differences in the visual stimuli—which were the same across conditions, see Fig. 4. At the individual level there was a large variability in the percentage of significant decoding across the time range of interest, ranging from 0.00 to 87.07% (M = 19.30 ± 23.92). This variability was not correlated with participants face expertise ($r=-.01$, 95% CI $\left[-.31,.29\right]$, $t\left(42\right)=-0.06$, $p=.951$). There was also no correlation between face expertise and individual peak level decoding nor its latency (*p* < 0.465).

When face stimuli were observed, contrary to what was originally predicted, we also observed above chance decoding of face expectations (high vs low) at the group level. Significant decoding of the neural response to the same visual input (faces) for this contrast started at 108 ms until 500 ms (almost uninterruptedly, see Fig. 4). As before, at the individual level, there was a large variability of percentage of significant decoding, ranging from 0.00% to 77.59% (M = 15.45 ± 21.97). Face expertise was not correlated with this variable measure ($r=-.06$, 95% CI $\left[-.33,.22\right]$, $t\left(48\right)=-0.41$, $p=.681$) nor with peak decoding level ($r=.08$, 95% CI $\left[-.21,.35\right]$, $t\left(48\right)=0.53$, $p=.597$). However, there was a trend for a correlation between face expertise and peak latency ($r=.24$, 95% CI $\left[-.04,.49\right]$, $t\left(48\right)=1.75$, $p=.087$; Supplementary Table 2 for results with raw CFMT+).

The results of the behavioural experiment suggest that face expectation effects are particularly relevant when participants observe faces, an effect that was linked to facial expertise. In response, we conducted an exploratory analysis to investigate the potential association between these behavioural measures and individual metrics of face expectation decoding when faces were observed. Critically, facilitation effects were correlated with latency of peak decoding ($r=-.30$, 95% CI $\left[-.53,-.03\right]$, $t\left(48\right)=-2.19$, $p=.033$; with no other significant correlation with decoding metrics *p* < 0.693), in a similar pattern as what was observed for face expertise. Interference effects associated with low face expectations were not correlated with any of the decoding metrics (*p* < 0.144). Importantly car expertise was not correlated with any decoding metrics (Supplementary Table 3).

## Discussion

In the current study, we set out to explore how the effects of expectation on visual processing are modulated by expertise. Across two experiments, we manipulated the probabilistic association of a colour cue and the following visual category, contrasting effects observed with visual categories of high vs lower expertise (faces vs cars). This allowed us to probe the role of expertise on the behavioural and neural correlates of expectancy effects on perception both at the category and at the individual level. At the behavioural level, effects of expectation were robustly present only when participants saw the category of higher expertise (i.e. faces), with both facilitation for expected faces and interference for unexpected faces. These results support the adaptive purpose of predictive coding, where perception is facilitated by expectation^27,57,58^. Nonetheless, they are in contrast with the original hypothesis, in which we predicted that face expectation (both facilitating face, and inhibiting car perception) effects would be larger than effects of expectation of a less expert category (car expectation, i.e. low face expectation, facilitating car, and inhibiting face perception). This finding is contrary to our original predictions, but not unique. A similar pattern of behavioural results contrasting faces and a non-expert object category (houses), has been found where correct expectation led to faster encoding of faces and buildings, with a trend for this facilitation to be larger for buildings^27^.

Here crucially we go further to show an association between expertise and expectation effects at the individual level. Facilitatory effects of high face expectation on rapid face categorization, were associated with each participant’s face-selective recognition ability, and detrimental effects of low face expectation (i.e. trials with high face surprise) were also significantly mitigated by face expertise. This suggests that at the functional level, both representational units and prediction error units (face surprise) are modulated by individual expertise. At the neural level, state of the art multivariate decoding analysis (MVPA) provides direct evidence of the role of expectation on early perception. Specifically, we identified significant decoding differences as a function of face expectation starting around 100 ms post stimulus in response to identical visual stimulation. This finding is in line with the results of previous fMRI studies that identify effects of expectation over several structures of the face network^25–28^ and for the first time provides an indication of the time course of such an effect. The present findings indicate that expectation has an impact on perceptual processing very early in the time course of face processing. We note that this neural sensitivity appears even earlier than the classic N170 component (occurring around 170 ms after image onset), which is often the first ERP component considered for face encoding^59^. Of relevance, at the individual level, high vs low face expectation decoding (peak latency) for observed faces, was significantly associated with expectation-driven facilitation of face categorization in the behavioural task. A marginal association was also observed with individuals’ face expertise (selective measure of face recognition ability) together suggesting that expectation related activity in posterior sensory areas has a functional role.

The observed links between behaviour and neural metrics are particularly notable when considering that the various tasks in the current study used different stimuli. This association indicates that the early neural signals of expectation observed over perceptual areas (i.e., posterior channels), are not only associated with specific exemplars used, but instead are generalised to the broader category predicted (i.e. faces). This highlights the adaptive role that expectation has on our ability to perceive and categorize stimuli in a flexible and timely manner.

Interestingly, it was later peak decoding of expectation in individuals that was associated with a greater behavioural facilitation and higher face expertise, suggesting that effects of expectation associated with face expertise are most pronounced later in the face processing timecourse (around 200 ms and after). This time window is associated with face identification and familiarity processing^60,61^ and ERP components occurring in this period have been previous linked with face expertise (N250, N250R, P300, P600^62–65^). The current findings might suggest that effects of face expertise emerging later in the neural timecourse, could be partially attributed to more expert participants’ ability to generate stronger expectations.

We found similar decoding of expectation for the expert and non-expert observed category. This result was contrary to our original predictions, which were motivated by previous work that identified expectation-related effects for faces but not for non-expert categories in fMRI^12,13,15^. Nevertheless it is in line with previous neural imaging findings, showing expectation driven selective cortical activation (fMRI) for both expert and non-expert categories in a similar paradigm (colour contingent face and house expectation^27^). Furthermore, in the current work, there is not a contrast where expectation of the expert condition is fully absent. Future work could more fully tease apart the role of expertise by contrasting two non expert categories (e.g. houses and cars), to establish what role, if any, expectation plays in the perception of such non expert categories. Moreover, while recognizing that humans have varying degrees of expertise in processing faces, we do not dismiss the possibility that faces may be considered an inherently privileged visual category due to their social relevance (for a review see^66^). Future work can further explore the importance of expertise, by selectively recruiting participants with varying degrees of expertise in a specific non face object (e.g. birds^67^).

Employing a decoding approach revealed effects of expectation and their association with behaviour that were not seen at the traditional N170 level, highlighting the exceptional sensitivity of MVPA techniques to differential patterns of neural activity that would otherwise not be captured with more conventional analysis pipelines. We note that the current experimental design and analysis cannot inform about the exact neural populations underlying the observed decoding, which could include neural activity from neural populations sensitive to both face and object processing. The use of the CFMT+ as an independent measure of face expertise allowed us to directly probe the role of expertise at the individual level, revealing its strong role at the functional level, as evidenced by the behavioural findings, and more tentatively indicating that its neural underpinnings might occur later in the timecourse. To further address the possibility that expectation processing might underpin some of the variability observed on face expertise at the population level, future studies could use famous faces, or personally familiar faces, to assess if expectation effects robustly modulate later ERP components associated with identity processing and face expertise.

In summary, our findings provide compelling evidence that expectation shapes very early perception, with significant decoding of the neural response to identical visual stimulation driven by expectation cues from 100 ms into the time course of processing. For objects of expertise (faces) we show a direct link at the individual level between behavioural facilitation effects of face expectation (faster response when faces are expected) and the latency of peak decoding of the neural response to faces when expected or not. Of note, expectation effects for face stimuli are also associated with independent standardized measures of face identification expertise at least robustly at the behavioural level. No such relationships with individual metrics are seen for the non-expert category (cars). Taken together, these results provide clear time-resolved verification of the influence of expectation on early perception, supporting a role of higher-level expertise in forming priors and highlighting the importance of the typically overlooked contribution of individual level variability.

### Supplementary Information

Supplementary Information.

## Data Availability

Code for analysis of the behavioural data and ERP analysis of the EEG data is available at https://osf.io/gmz8k/. The code underpinning the MVPA analysis (which was fine-tuned for the present work) is available at https://github.com/fws252/Mares_etal_Cortex_2022. Raw data from consenting participants is available online at Birkbeck Research Data Repository (https://researchdata.bbk.ac.uk/id/eprint/214/*)*, data from the remaining participants (N = 10) can be obtained upon request to the corresponding author (IM). For the purposes of open access, the authors have applied a CC BY public copyright licence to any author accepted manuscript version arising from this submission.
