# Supplementary material for: Effects of expectation on face perception and its association with expertise
Source: Sci Rep. 2024 Apr 24;14:9402. doi: 10.1038/s41598-024-59284-0 (PMC11043383; doi:10.1038/s41598-024-59284-0)
Supplement: Supplementary file 1 — Supplementary Information. [file 41598_2024_59284_MOESM1_ESM.docx]

# Supplementary Material


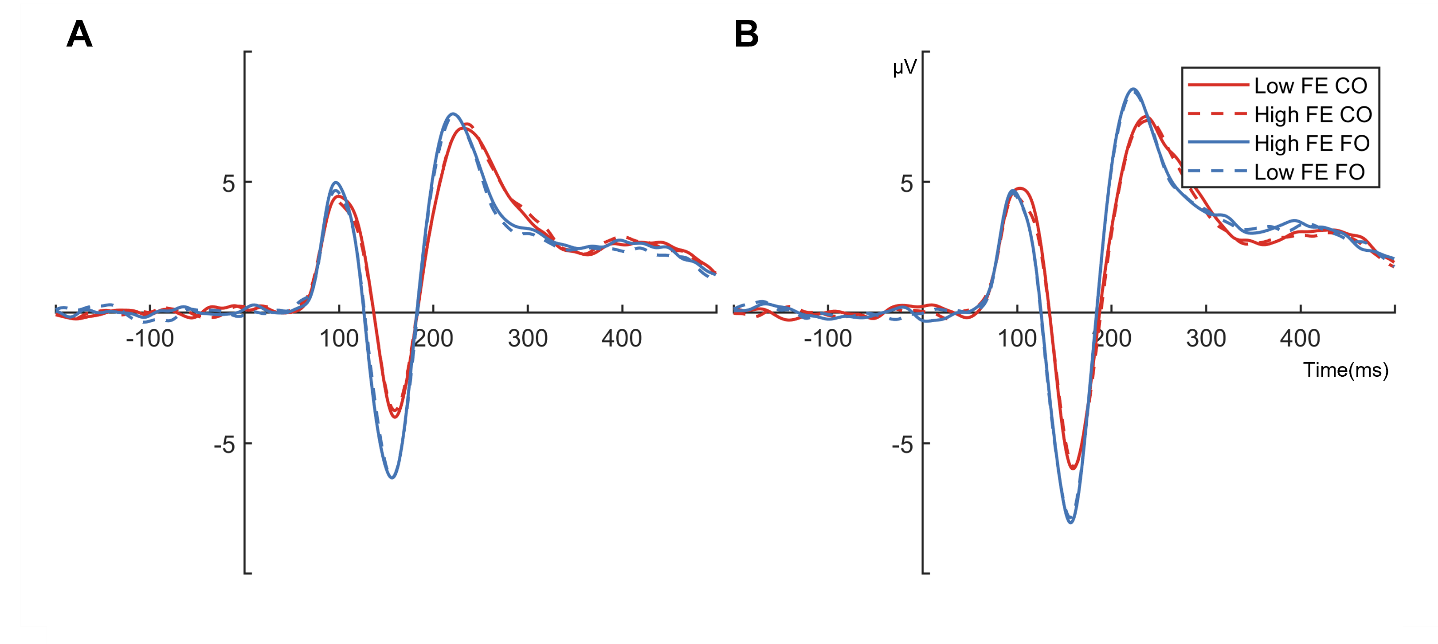


S Figure 1 - ERPs waveforms over the left hemisphere in panel A (P7/PO7) and the right hemisphere (P8/PO8); FE – Face Expectation; CO – Car Observed; FO – Face observed.

S Table 1:

Summary of correlation of the behavioural effects observed with raw scores of the CFMT.

|  | *p* | *r* | Confidence Interval |
| --- | --- | --- | --- |
| High face expectation, face seen | .016 | -.31 | [-.53, -.06] |
| Low face expectation, face seen | .012 | -.33 | [-.54, -.08] |
| No expectation, face seen | .802 | .03 | [-.22, .29] |

S Table 2:

Summary of correlation of the MVPA measures with raw scores of the CFMT.

|  | *p* | *r* | Confidence Interval |
| --- | --- | --- | --- |
| High vs Low face expectation, car seen - % | .860 | -.03 | [-.32, .27] |
| High vs Low face expectation, car seen - Peak decoding | .691 | -.06 | [-.35, .24] |
| High vs Low face expectation, car seen - Peak latency | .576 | .09 | [-.22, .37] |
| High vs Low face expectation, face seen - % | .707 | -.05 | [-.33, .23] |
| High vs Low face expectation, face seen - Peak decoding | .573 | .08 | [-.20, .35] |
| High vs Low face expectation, face seen - Peak latency | .098 | .24 | [-.04, .48] |

S Table 3:

Summary of correlation of the MVPA measures with CCMT.

|  | *p* | *r* | Confidence Interval |
| --- | --- | --- | --- |
| High vs Low face expectation, car seen - % | .300 | -.16 | [-.44, .14] |
| High vs Low face expectation, car seen - Peak decoding | .728 | .05 | [-.25, .35] |
| High vs Low face expectation, car seen - Peak latency | .109 | -.25 | [-.51, .06] |
| High vs Low face expectation, face seen - % | .754 | .05 | [-.24, .32] |
| High vs Low face expectation, face seen - Peak decoding | .741 | .05 | [-.23, .32] |
| High vs Low face expectation, face seen - Peak latency | .643 | -.07 | [-.34, .22] |
